# A Polyphenol-Rich Olive Oil Byproduct-Derived Nutraceutical Preserves Muscle Health in Adults at Metabolic Risk: A Secondary Analysis of a Pilot Study

**DOI:** 10.3390/nu18101551

**Published:** 2026-05-14

**Authors:** Danilo Morelli, Sara Nofri, Paola Corradino, Domenico E. Pellegrini-Giampietro, Calogero Caruso, Anna Aiello, Adriana Albini

**Affiliations:** 1 Fondazione IRCCS Istituto Tumori di Milano, 20133 Milan, Italy; danilo.morelli@istitutotumori.mi.it; 2 Department of Health Sciences, Section of Clinical Pharmacology and Oncology, University of Florence, 50139 Florence, Italy; sara.nofri@googlemail.com (S.N.); domenico.pellegrini@unifi.it (D.E.P.-G.); 3 European Institute of Oncology (IEO), Istituto di Ricovero e Cura a Carattere Scientifico (IRCCS), 20141 Milan, Italy; paola.corradino@ieo.it; 4 Laboratory of Immunopathology and Immunosenescence, Department of Biomedicine, Neuroscience and Advanced Diagnostics, University of Palermo, 90133 Palermo, Italy; calogero.caruso@unipa.it (C.C.); anna.aiello@unipa.it (A.A.); 5 Scientific Directorate, European Institute of Oncology (IEO), Istituto di Ricovero e Cura a Carattere Scientifico (IRCCS), 20141 Milan, Italy

**Keywords:** muscle health, prevention, sarcopenia, cancer, olive oil, polyphenols, hydroxytyrosol, nutraceutical

## Abstract

**Background**: Muscle function determines overall health and is often impaired in metabolic syndrome and cancer, largely due to oxidative stress and inflammation. Olive mill wastewater (OMWW) is rich in bioactive polyphenols (e.g., hydroxytyrosol and verbascoside) that may hinder these potential pro-sarcopenic mechanisms, representing a potential nutraceutical to limit muscle health decline. **Objective**: To evaluate the effects of short-term supplementation with an OMWW-derived polyphenol extract (Oliphenolia^®^, OMWW-OL) on muscle-related parameters and antioxidant biomarkers in adults at metabolic risk while maintaining dietary habits. **Methods**: This exploratory, hypothesis-driven secondary analysis was based on a single-arm longitudinal pilot study assessing patients at baseline (T0), after 30 days of supplementation (T1), and 30 days post-discontinuation (T2). Anthropometry, bioelectrical impedance, and biochemical assessments were performed. **Results**: Supplementation was associated with modest increases in skeletal muscle mass, muscle mass percentage, and wrist, arm, and calf circumferences. Fat mass decreased progressively, while total body water percentage and hydration status improved. Ferritin levels rose at T2, alongside increases in protein thiols (PSH) and Trolox equivalent antioxidant capacity (TEAC), suggesting improved iron status and reduced oxidative stress. Body weight and BMI decreased, as expected in a dietary intervention for metabolic syndrome, while muscle health showed a tendency toward improvement. **Conclusions**: Although the findings require cautious interpretation, short-term OMWW-OL supplementation was associated with modest but consistent directional changes in muscle-related and metabolic indicators in adults at metabolic risk. The results support hypothesis generation and highlight the need for larger studies to further explore the potential role of OMWW-OL in the context of cancer-associated sarcopenia.

## 1. Introduction

An expanding body of evidence indicates that muscular health is inversely and independently associated with both all-cause and cardiovascular mortality, even after adjustment for cardiorespiratory fitness and other confounding factors, including age, adiposity, and smoking status. Multiple studies have demonstrated that greater muscular performance is associated with a lower risk of several chronic conditions, including cardiovascular disease and stroke [1,2], hypertension [3], metabolic syndrome and hyperinsulinemia [4,5], and type 2 diabetes [6]. Conversely, metabolic syndrome has been consistently associated with declines in muscle function and represents a significant contributor to the development of sarcopenia [7], a progressive condition characterized by reductions in muscle mass, strength, and physical performance [8]. Notably, dyslipidemia has also been associated with reduced muscle strength and impaired physical performance [9]. Pathophysiological mechanisms linking metabolic dysfunction to muscle deterioration include insulin resistance, chronic low-grade inflammation, oxidative stress, and ectopic fat deposition, all of which impair protein synthesis and mitochondrial function, thereby accelerating muscle mass and functional decline [10,11,12]. In particular, oxidative stress plays a major role in muscle mass loss, as reactive oxygen species (ROS) regulate key signaling pathways involved in muscle metabolism, adaptation, and regeneration. Consequently, when redox balance is disrupted, such as in metabolic syndrome, excess ROS triggers inflammation, creating an environment that promotes protein degradation, impairs mitochondrial function, and reduces regenerative capacity. This leads to muscle dysfunction and decline [13], which is especially pronounced in metabolic syndrome [14]. Declines in muscle function also frequently occur secondary to malnutrition, physical inactivity, cancer, or prolonged immobilization [15] and represents a major clinical concern, as they are associated with an increased risk of mortality [16,17]. Therefore, strategies aimed at preserving muscle mass and function are essential [18]. Nutritional deficiencies, including inadequate protein intake, vitamin D deficiency, and low antioxidant consumption, further compromise muscle regeneration and metabolism [19,20], while low serum albumin levels may serve as an early biomarker of muscle-wasting risk [21]. Although resistance exercise, adequate nutrition, and hormonal modulation can partially restore muscle function [22], effective pharmacological therapies for sarcopenia remain limited [23,24].

Preserving muscle function, particularly strength and physical performance, is therefore crucial. Early preventive strategies targeting metabolic dysfunction, oxidative stress, and inflammation may play a key role in supporting muscle integrity, functional independence, and long-term health in at-risk populations.

In this context, increasing attention has focused on nutraceutical approaches [25], among which olives and olive oil stand out due to their content in polyphenols [26,27]. Interestingly, olive mill wastewater (OMWW), a byproduct of olive oil extraction once considered solely an environmental pollutant, also contains bioactive polyphenols such as hydroxytyrosol and verbascoside, which exhibit well-documented antioxidant and anti-inflammatory properties [28,29,30]. The valorization of OMWW through biorefinery approaches supports circular economy principles by transforming agro-industrial waste into sustainable nutraceutical ingredients. Its polyphenol-rich composition makes OMWW-derived extracts promising candidates for supporting muscle health, and a potential anti-sarcopenic effect has been suggested [31]. Consistently, preclinical evidence indicates that hydroxytyrosol may enhance mitochondrial function and protect against muscle atrophy [32,33,34]. In addition, in vitro studies have reported reductions in intestinal cell inflammation and modulation of immune responses [35], while in vivo experiments suggest that OMWW-derived extracts may reduce chemotherapy-induced cardiotoxicity [36]. Taken together, these findings indicate that OMWW polyphenols may provide systemic benefits, supporting muscle health, intestinal and immune function, and cardiovascular protection. These effects may be particularly relevant not only for the general population but also for individuals at risk of metabolic syndrome, while their applicability to conditions such as cancer-associated sarcopenia or to patients undergoing cancer treatment requires dedicated investigation.

The present study represents a secondary, hypothesis-driven re-analysis of data generated within a single-arm longitudinal interventional pilot trial originally designed to investigate the metabolic and anti-inflammatory effects of an OMWW-derived polyphenol extract (Oliphenolia^®^, OMWW-OL) [29]. OliPhenolia^®^ is a polyphenol- and hydroxytyrosol-rich phytocomplex obtained from OMWW through concentration, reverse osmosis, and mechanical filtration processes [37]. Although the primary analysis focused on cardiometabolic outcomes, the original dataset included repeated anthropometric, bioelectrical impedance, and functional measurements relevant to muscle strength and body composition. This re-analysis therefore specifically evaluated the effects of standardized OMWW-OL supplementation on muscle-related parameters in adults at risk of developing metabolic syndrome, with the aim of exploring potential associations with muscle function and body composition over time. The novelty of this study lies in its shift from predominantly preclinical and general metabolic research on OMWW-OL supplementation to a human, hypothesis-driven analysis specifically targeting muscle health, integrating body composition, anthropometric, hydration, and antioxidant markers to position OMWW-OL within the emerging framework of early support for muscle health and resilience.

## 2. Methods

### 2.1. Study Design and Participants

The original study was a single-arm, longitudinal, interventional pilot study conducted in western Sicily within the research program Nutraceutical Effects of Olive Products: Role in the Achievement of Longevity to evaluate the effects of an olive mill wastewater-derived polyphenol extract (Oliphenolia^®^, OMWW-OL) on anthropometric and hematological parameters. No new studies were conducted, and no additional intervention was performed. The paper presents a re-analysis of previously collected data. In this design, each participant served as his/her own control. Adults presenting with at least one metabolic syndrome-related characteristic were enrolled. Inclusion criteria of the original study comprised mild dyslipidaemia (total cholesterol 190–240 mg/dL or triglycerides ≥ 150 mg/dL), increased waist circumference (≥102 cm in men or ≥88 cm in women), or impaired fasting glucose (≥100 mg/dL). Exclusion criteria included chronic systemic disease, ongoing pharmacological treatment for metabolic disorders (e.g., statins or hypoglycaemic agents), adherence to restrictive diets, or prior use of polyphenol supplements [29].

### 2.2. Objectives and Analytical Hierarchy

This study represents a secondary, hypothesis-driven re-analysis of data derived from a previously conducted single-arm pilot trial. The present analysis was exploratory and examined data not analyzed in the original paper. Muscle-related outcomes were selected a priori based on the availability of repeated-measures and their clinical relevance to muscle mass. The primary exploratory endpoints of this secondary analysis included changes in body composition parameters, such as skeletal muscle mass (SMM), skeletal muscle index (SMI), fat mass (FM) and muscle mass (MM), as well as changes in anthropometric measures, hydration indices, and biochemical markers over time (T0, T1, and T2). Given the exploratory nature of this pilot re-analysis, no formal adjustment for multiplicity was applied. Therefore, all statistical findings should be interpreted as hypothesis-generating.

### 2.3. Ethical Aspects

All participants in the original study were thoroughly informed beforehand about the study’s objectives and procedures and provided written informed consent prior to enrollment. To ensure confidentiality, personal identifiers were replaced with coded alphanumeric labels in compliance with the European General Data Protection Regulation (GDPR, EU 2016/679). The study was carried out according to the principles of the Declaration of Helsinki (1964) and its subsequent revisions. Ethical approval was granted by the Institutional Review Board of the Policlinico Paolo Giaccone University Hospital (study title: role of olive products in the prevention of age-related diseases) on 18 February 2019 under number 02/2019.

### 2.4. Intervention

Following a seven-day washout period during which participants abstained from extra virgin olive oil and polyphenol-containing diet or supplements, subjects consumed 25 mL of OMWW extract (Oliphenolia^®^) twice daily, 30 min before lunch and dinner, for 30 days. Oliphenolia^®^ was provided by Fattoria La Vialla di Gianni, Antonio e Bandino Lofranco SAS (Castiglion Fibocchi, Arezzo, Italy). Among the identified polyphenols, hydroxytyrosol was the most abundant, representing about 18.3% (82.50 mg), followed by verbascoside at 7.2% (32.50 mg), tyrosol at 2.3% (10.50 mg), and p-coumaroyl secoiridoids at 1.4% (6.50 mg; Table 1) [38].

### 2.5. Assessments

Muscular function and sarcopenia-related variables were selected a priori based on international consensus frameworks, principally the European Working Group on Sarcopenia in Older People (EWGSOP2) [8], with consideration of complementary guidance from the Sarcopenia Definitions and Outcomes Consortium (SDOC) [31] and the Asian Working Group for Sarcopenia (AWGS) [32].

Calf circumference was included as a complementary anthropometric proxy of lower-limb muscle mass and muscle strength. Arm and wrist circumferences were also measured. Measurements were obtained at the point of maximal girth with participants standing and muscles relaxed. These parameters are recommended in community and field settings where imaging- or performance-based assessments are not feasible, and they have demonstrated strong correlations with appendicular muscle mass and strength. Muscle quantity and body composition were evaluated using bioelectrical impedance analysis (BIA), a validated, non-invasive, and reproducible method suitable for longitudinal and nutraceutical studies when hydration status and standardized testing conditions are controlled. SMM, FM, MM, and SMI were assessed. Hydration-related parameters derived from BIA, including total body water and hydration status, were also examined, as they reflect cellular integrity and may influence muscle function and bioimpedance-based estimates. In addition, biochemical parameters were measured in blood samples [29].

### 2.6. Data Collection

All measurements were obtained at three predefined time points: baseline (T0), after 30 days of OMWW supplementation (T1), and 30 days after discontinuation of supplementation (T2). This design enabled evaluation of both short-term intervention effects and persistence following washout. Anthropometric, clinical, and BIA analyses were conducted under standardized conditions by trained biologists and physicians. Fasting blood samples were collected at each time point for the assessment of hematochemical parameters.

BIA (Akern 101), which has been validated as a practical and reliable tool for sarcopenia assessment [39], was used to estimate SMM (kg), SMI, (kg/m^2^), MM (kg), FM (kg), total body water (TBW) and hydration status. Anthropometric measurements and BIAs were performed with participants wearing light clothing and barefoot. Body weight was measured using a calibrated electronic scale (in kilograms), while height was assessed in the supine position using a stadiometer. BIA measurements also included resistance. Body composition parameters, expressed both as percentages of body weight and in kg/m^2^, were estimated using regression equations implemented in Bodygram^®^ Plus 1.1.4.4 software (BIA-101, RJL Systems, Akern, Pisa, Italy). Blood samples were collected in the morning after an overnight fast into additive-free tubes for serum separation and subsequent measurement of ferritin, renal and hepatic function parameters, and inflammatory markers. Samples were subsequently centrifuged at 2500 rpm for 15 min at 4 °C, and the isolated serum was frozen and stored at −80 °C. Standard biochemical assays were used to assess all hematochemical parameters. For protein thiols (PSH) and Trolox equivalent antioxidant capacity (TEAC) measurements, blood samples were collected in heparinized gel-separation vacutainer tubes and centrifuged at 3000 rpm (500× *g*) for 5 min. Plasma was carefully separated and stored at −18 °C. PSH concentrations were measured using a standardized Ellman’s assay, with calibration and absorbance readings obtained at 412 nm [40]. TEAC was measured according to a previously reported protocol [41]. Clinical and biochemical safety parameters and adverse events were monitored throughout the study period.

### 2.7. Statistics

The present analysis was conducted on a per-protocol basis, including only participants who completed all three assessment time points (T0, T1, and T2) [29]. An intention-to-treat approach was not applied due to the exploratory nature of the study and the limited sample size. No imputation of missing data was performed. Statistical evaluation focused on within-participant longitudinal comparisons across time points (T0, T1, and T2). Calculations of mean values, standard deviations (SDs), and average percentage changes between time points (T0–T1, T1–T2, and T0–T2) were performed using Excel.

Statistical analyses were performed using GraphPad Prism version 10.0.2 (GraphPad Software, San Diego, CA, USA). Changes across time points (T0, T1, and T2) were analyzed using one-way repeated-measures analysis of variance (ANOVA), with time as the within-subject factor. When appropriate, Tukey’s multiple comparisons post hoc test was applied to assess pairwise differences between time points (T0 vs. T1, T0 vs. T2, and T1 vs. T2), and adjusted *p*-values are reported. Results are presented as mean ± SD. A two-sided *p*-value < 0.05 was considered statistically significant. Non-significant variations were descriptively reported as percentages of change. Given the number of endpoints assessed and the absence of correction for multiple comparisons, reported *p*-values should be considered nominal and interpreted cautiously.

## 3. Results

### 3.1. Baseline Characteristics of Enrolled Participants

All the characteristics of enrolled participants were reported in the original study manuscript [29]. Twenty-nine adults (seventeen men and twelve women) with at least one metabolic syndrome-related characteristic were enrolled in the study, and twenty-three participants (fifteen men and eight women) completed all three assessment time points: baseline (T0), after 30 days of OMWW-OL supplementation (T1), and 30 days after discontinuation (T2). Six participants withdrew for personal reasons unrelated to adverse events. The study cohort consisted of middle-aged to older adults, with a mean age of 59 years at baseline. Anthropometric and muscle parameters indicated a population with characteristics potentially associated with metabolic and muscle-related alterations. Mean weight was 76.55 kg and BMI was 27.46 kg/m^2^. Body composition analysis revealed FM of 23.31 kg, MM of 24.71 kg, SMM of 24.71 kg, and SMI of 8.77 kg/m^2^. Relative composition showed FM% of 30.03% and MM% of 32.41%. Mean resistance was 548.26 Ohms. Physical assessment indicated a calf circumference of 34.70 cm, wrist circumference of 16.46 cm and arm circumference of 29.00 cm. TBW was 39.09 L, TBW% was 51.36% and hydration status was 73.40%. Ferritin levels were 108.48 ng/mL, PSH levels were 4.79 µmol/L and TEAC levels were 4380.09 mmol/L. Biochemical parameters were largely within normal or near-reference ranges. Alanine aminotransferase (ALT) was 21.57 U/L, and aspartate aminotransferase (AST) was 18.39 U/L. Gamma-glutamyl transferase (GGT) levels averaged 25.52 U/L, while kidney function markers included urea at 34.60 mg/dL and creatinine at 0.84 mg/dL.

Overall, participants presented baseline characteristics consistent with older adults at risk of metabolic alterations, with no evidence of muscle decline and moderate metabolic risk factors.

### 3.2. Longitudinal Changes in Body Composition: Lean Mass Preservation with Progressive Fat Mass Reduction

**Figure 1:**
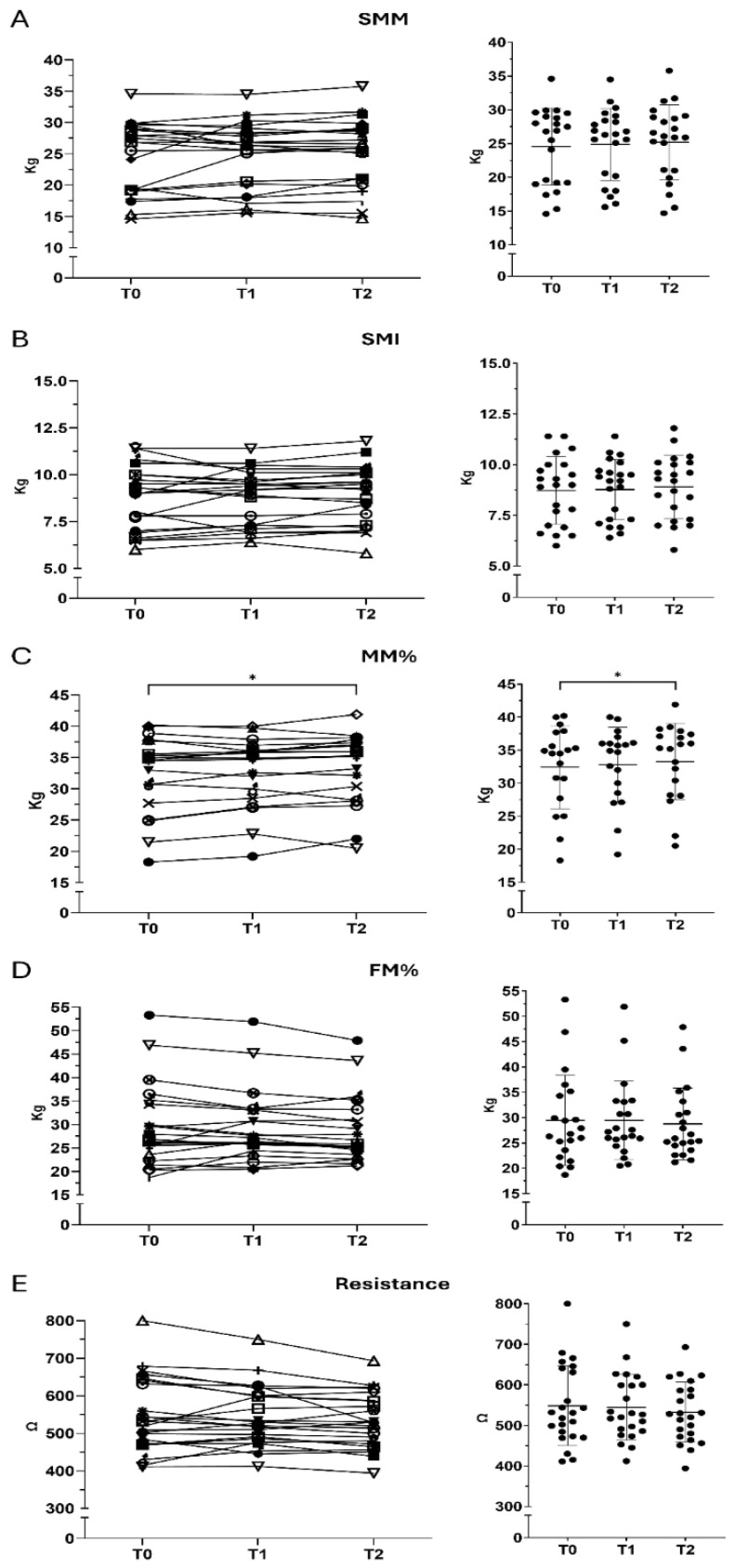
Longitudinal changes in body composition parameters assessed by bioelectrical impedance analysis (BIA). BIA-derived body composition parameters measured at baseline (T0), post-supplementation (T1), and 30 days after cessation (T2). (**A**) Skeletal muscle mass (SMM). (**B**) Skeletal muscle index (SMI). (**C**) Muscle mass % (MM%). (**D**) Fat mass percentage (FM%). (**E**) Resistance. Data are presented as mean ± SD. Statistical significance was assessed using repeated-measures ANOVA with Tukey’s post hoc test. * *p*-value < 0.05.

BIA revealed modest but broadly consistent directional changes in body composition following supplementation over the study period. Overall, body composition parameters remained largely stable, with slight shifts toward lean mass proportions. FM showed consistent reductions over time, decreasing by 4.3% from T0 to T2. FM% also showed a progressive decline over time. Mean values decreased from T0 to T1 and further at T2 (−3.5% compared with baseline). Similar trends were observed for FMI, which declined by 4.2% at T2. In contrast, the proportional measures of lean tissue showed slight increases. MM% showed a modest increase over time. Mean values increased slightly from T0 to T1 and further at T2 (+2% compared with baseline, *p* = 0.0417). Between T1 and T2, MM% increased by an additional 1.5%, possibly suggesting a long-lasting effect. SMM remained stable across time points. A minimal increase was observed at T2 (+1.0% compared with baseline). Between T1 and T2, SMM increased by approximately 1.2%. SMI showed minimal variation over time. A slight increase was observed at T2 (+1.0% vs. T0). Between T1 and T2, SMI increased by approximately 1.5%. These findings indicate overall stability, with a slight upward trend by the end of follow-up. Resistance showed a slight reduction from T0 to T1 (−0.7%), followed by a more pronounced decline at T2 (−2.9% vs. T0). Between T1 and T2, resistance decreased by an additional −2.2%. Overall, resistance showed a downward trend over time (Figure 1).

### 3.3. Longitudinal Changes in Peripheral Anthropometric Parameters

**Figure 2:**
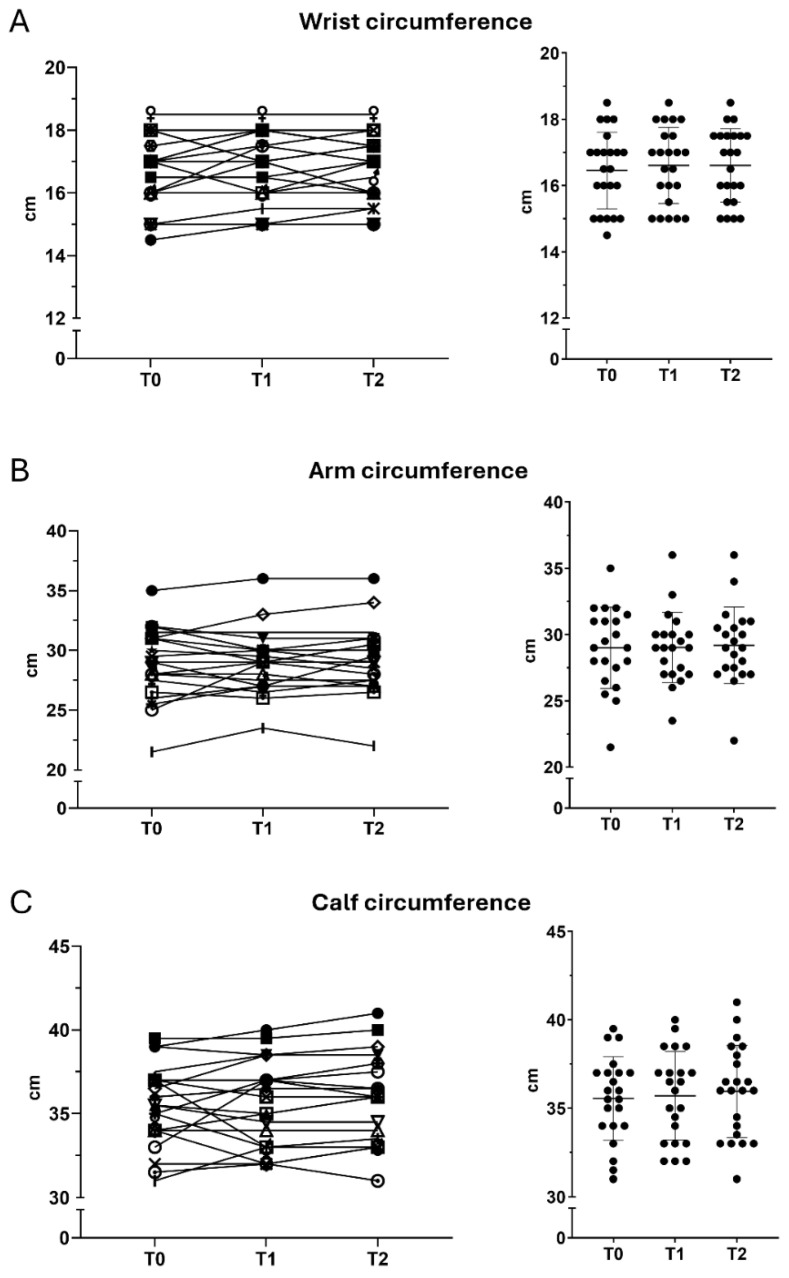
Changes in anthropometric circumferences. Anthropometric parameters evaluated at T0, T1, and T2. (**A**) Wrist circumference, (**B**) arm circumference, and (**C**) calf circumference. Values are expressed as mean ± SD. Comparisons across time points were performed using repeated-measures ANOVA with Tukey’s correction.

Arm circumference showed a slight decrease, suggesting overall stability. Calf circumference showed a progressive increase over time. Compared with baseline, values increased slightly at T1 (+0.3%) and more substantially at T2 (+3.6%). Between T1 and T2, an additional 3.3% increase was observed. Wrist circumference increased modestly from T0 to T1 (+0.9%) and remained stable thereafter, with no further change at T2 (Figure 2).

### 3.4. Longitudinal Changes in Total Body Water and Hydration Status

**Figure 3:**
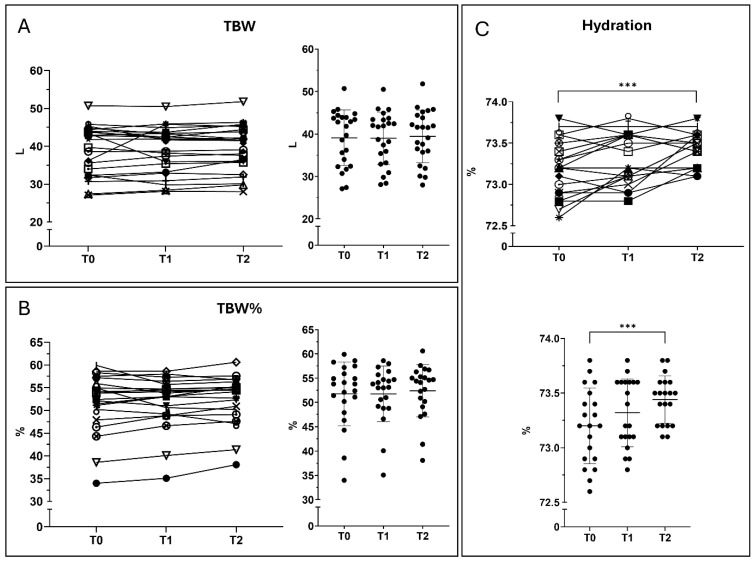
Hydration and total body water parameters. Hydration-related parameters derived from BIA measured at T0, T1, and T2. (**A**) Total body water (TBW, L). (**B**) Total body water percentage (TBW%). (**C**) Hydration status (%). Data are shown as mean ± SD. Statistical analysis was performed using repeated-measures ANOVA with Tukey’s multiple comparisons test. *** *p*-value < 0.001.

TBW remained stable throughout the study period. A modest increase was observed at T2 (+1.0% vs. baseline). Between T1 and T2, TBW increased by approximately 1.1%, suggesting overall stability of hydration status. TBW% showed a progressive increase over time. Compared with baseline, TBW% rose by 0.4% at T1 and 1.7% at T2. Between T1 and T2, a further increase of 1.3% was observed. These findings are consistent with a modest directional change in body composition parameters. Hydration values showed a small but progressive increase over time. A slight, non-significant rise was observed from T0 to T1 (+0.16%). Hydration increased further at T2, and the difference between T0 and T2 reached statistical significance (*p* = 0.0002), indicating a subtle yet consistent improvement in hydration status across the study period (Figure 3).

### 3.5. Longitudinal Changes in Ferritin, Protein Thiols (PSH), and Antioxidant Capacity

**Figure 4:**
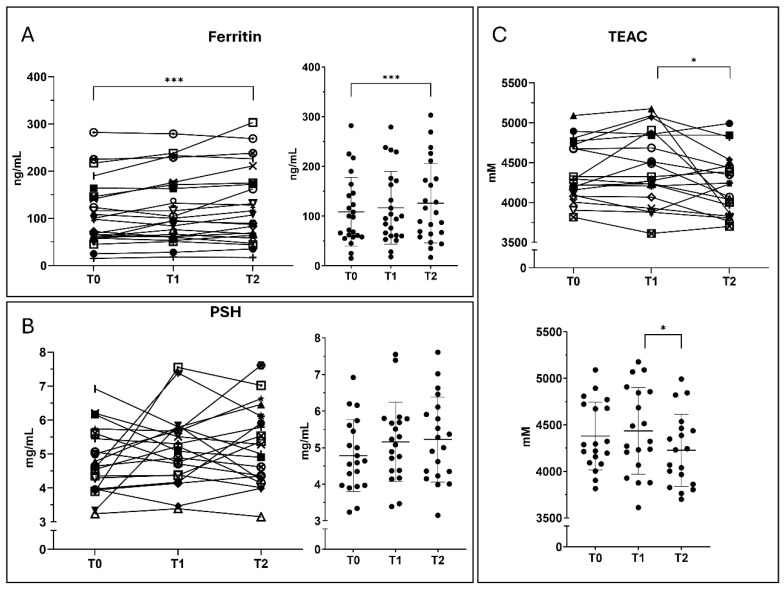
Iron stores and antioxidant biomarkers. Longitudinal changes in biochemical markers related to iron metabolism, redox balance and antioxidant capacity. (**A**) Ferritin (ng/mL). (**B**) Protein thiols (PSH, μmol/L). (**C**) Trolox equivalent antioxidant capacity (TEAC). Measurements were obtained at T0, T1, and T2. Data are presented as mean ± SD. Statistical significance was assessed using repeated-measures ANOVA with Tukey’s post hoc analysis. * *p*-value < 0.05; *** *p*-value < 0.001.

Ferritin levels showed a progressive increase over time. A moderate, non-significant rise was observed from T0 to T1 (+7.7%). Ferritin increased further at T2, reaching a +16.2% change compared with baseline, and the difference between T0 and T2 was statistically significant (*p* = 0.0007). Between T1 and T2, a further 7.9% increase was observed. PSH levels also showed a progressive increase over time. Compared with baseline, PSH increased by 7.7% at T1 and 9.0% at T2. Between T1 and T2, a smaller additional increase of 1.2% was observed. TEAC showed variable changes over time. Mean values increased from T0 to T1 and decreased at T2. No significant differences were observed between T0 and T1 or between T0 and T2, while a statistically significant difference was observed between T1 and T2 (*p* = 0.0221). These findings may reflect a directional change in antioxidant-related parameters (Figure 4).

### 3.6. Longitudinal Changes in Weight and BMI

**Figure 5:**
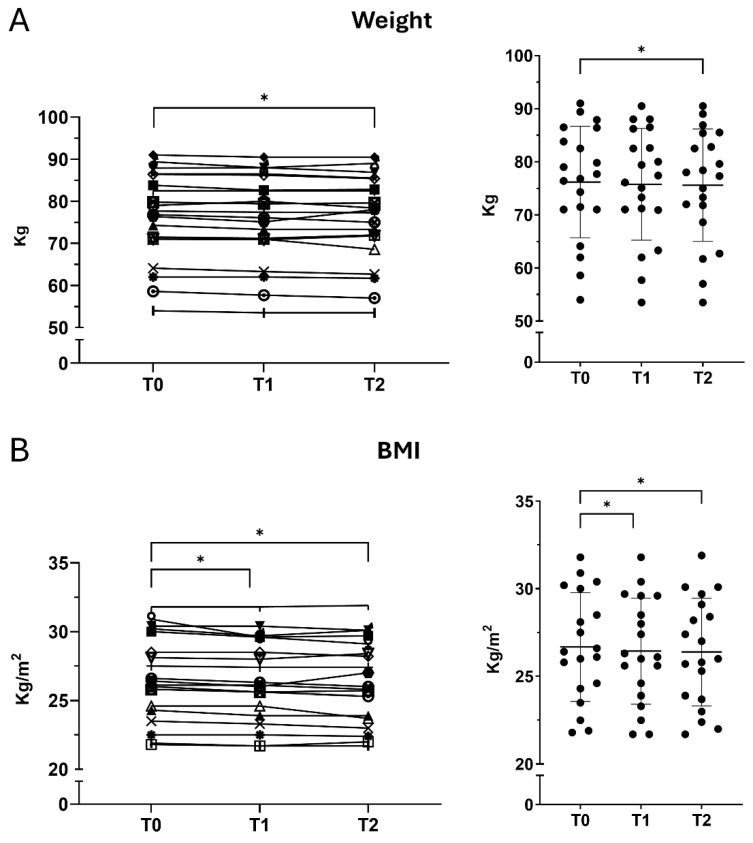
Body weight and body mass index. Changes in anthropometric measures over time. (**A**) Body weight (kg). (**B**) Body mass index (BMI, kg/m^2^). Values are expressed as mean ± SD at T0, T1, and T2. Statistical comparisons were conducted using repeated-measures ANOVA with Tukey’s multiple comparisons test. * *p*-value < 0.05.

Body weight showed a modest reduction over time. A slight decrease was observed from T0 to T1 (−0.7%). Weight declined further at T2 (−0.9% vs. baseline), and the difference between T0 and T2 reached statistical significance (*p* = 0.0097). BMI decreased progressively from T0 to T1 (−0.7%) and T2 (−0.9%), indicating a small but statistically significant steady reduction in body weight relative to height (*p* = 0.0097; Figure 5).

## 4. Discussion

As the global population ages [42], the prevalence and clinical burden of muscle-wasting syndromes are expected to increase [43]. Muscle mass and strength are key predictors of functional capacity and adverse outcomes [8,44,45]. Consequently, there is a growing need for preventive and multidisciplinary strategies. Oxidative stress represents a key therapeutic target, as it triggers inflammation, a major driver of muscle decline, particularly in the context of metabolic syndrome, which has been consistently associated with declines in muscle function [13,14]. Therefore, early interventions targeting metabolic dysfunction, oxidative stress, and inflammation may help preserve muscle integrity, functional independence, and long-term health in at-risk populations. To date, no interventional strategy has demonstrated sufficient and consistent clinical benefit to warrant routine use in this setting [46,47]. Consequently, attention has increasingly shifted toward nutraceutical approaches with antioxidant and anti-inflammatory properties, including polyphenol-rich extracts such as hydroxytyrosol derived from OMWW [48]. However, clinical evidence supporting their efficacy remains limited.

In this context, the present secondary analysis represents an exploratory, hypothesis-generating re-evaluation of muscle-related outcomes following short-term supplementation with an OMWW-derived polyphenol extract (Oliphenolia^®^, OMWW-OL) in adults at risk of metabolic alterations. Although originally designed to assess cardiometabolic parameters, the dataset provided a valuable opportunity to evaluate effects on skeletal muscle mass, body composition, hydration status, and oxidative biomarkers. While most changes did not reach statistical significance, likely due to the pilot design and limited sample size, overall, the results showed some consistent directional trends across several parameters.

The principal finding of this re-analysis is the consistent directional trend toward preservation and improvement in muscle composition over the study period, with several effects persisting even after a 30-day washout period. The internal coherence across anthropometric, bioimpedance, and biochemical parameters supports a biologically plausible effect. BIA-derived body composition parameters remained largely stable over time. Small directional reductions in fat mass and minimal increases in relative lean mass indices were observed. These changes should be interpreted cautiously but are indicative of compositional shifts. Importantly, improvements in muscle indices were accompanied by reductions in body weight and BMI. In people with pre-metabolic syndrome (often overlapping with metabolic syndrome risk factors), reductions in body weight and BMI are desirable when evaluating dietary supplementation. Weight and BMI reduction are typically associated with improved insulin sensitivity, lower fasting glucose, and a better lipid profile (decrease in triglycerides and increase in high-density lipoprotein [HDL]), all of which were assessed and described in the primary analysis [29], as well as lower cardiovascular risk. Therefore, if a supplement contributes to weight/BMI reductions, this is usually interpreted as a beneficial metabolic outcome.

Resistance showed a progressive decrease, which in BIA models is usually interpreted as being consistent with improved lean tissue conductivity. Lower muscle resistance post-supplementation is generally associated with improved muscle function, including a greater ability to contract and relax efficiently, reduced internal friction, and more coordinated movement. Collectively, these changes translate into improved performance and less fatigue [49].

Hydration-related measures showed modest but indicative changes over time, which may provide some contextual support for this interpretation. Total body water remained stable, while its percentage increased modestly over time.

The preservation of muscle-related parameters observed with OMWW-OL supplementation may suggest a potentially favorable metabolic profile. Hampering skeletal muscle mass decline is crucial not only for preventing physical frailty and disability but also for improving metabolic outcomes and reducing mortality risk [50].

Biochemical findings provide additional context. The increases in PSH and antioxidant capacity (TEAC) suggests enhanced antioxidant defenses, while the rise in ferritin within physiological ranges may reflect improved iron availability and metabolic homeostasis. Together, these changes are consistent with a systemic environment more conducive to muscle maintenance. Mechanistically, this aligns with the known antioxidant and anti-inflammatory properties of OMWW-derived polyphenols [28,29], which may modulate pathways implicated in muscle degradation. From a clinical perspective, attenuating skeletal muscle mass decline is essential for preventing frailty, improving metabolic outcomes, and reducing mortality risk [50]. These preliminary findings suggest that OMWW-derived polyphenols may support early preventive strategies, given the central role of oxidative stress and inflammation in muscle degradation and mitochondrial dysfunction [13,14]. Although these findings do not support clinical application at this stage, these results may provide a mechanistic rationale consistent with their known antioxidant and anti-inflammatory properties [28,29], thereby warranting further investigation.

**Figure 6:**
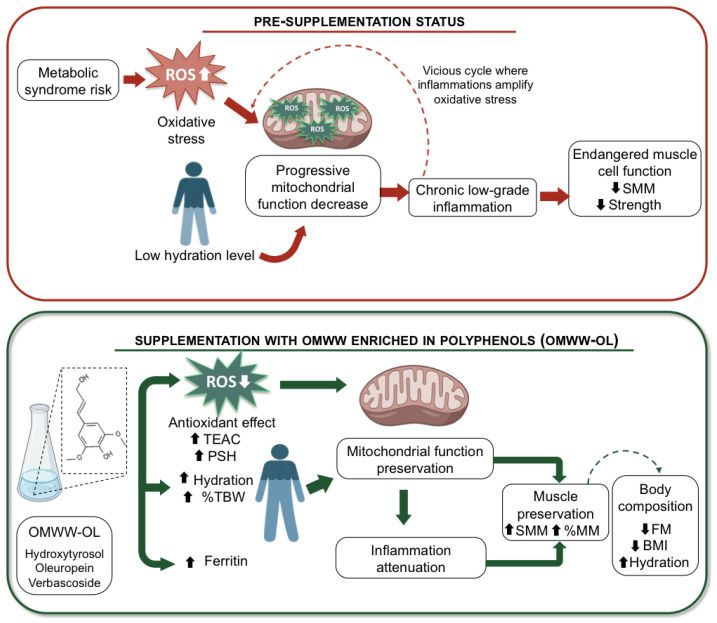
Proposed mechanism linking olive mill wastewater enriched in polyphenols (OMWW-OL) to muscle health. The upper panel illustrates the pathophysiological cascade associated with a diet not enriched in olive oil polyphenols. Increased metabolic syndrome risk leads to elevated reactive oxygen species (ROS) production and oxidative stress, contributing to mitochondrial damage in muscle cells. These alterations promote chronic low-grade inflammation, which in turn further increases oxidative stress, thereby perpetuating a vicious cycle that ultimately results in muscle decline characterized by reduced skeletal muscle mass (SMM) and strength. The lower panel depicts the potential beneficial effects of supplementation with OMWW-OL. Polyphenols (e.g., hydroxytyrosol and verbascoside) reduce ROS levels and oxidative stress (increased PSH and TEAC), improve hydration status and ferritin levels, and preserve mitochondrial function. The reduction in oxidative stress leads to attenuation of inflammation and supports the preservation of muscle mass and quality (increased SMM and %MM), as well as improvements in body composition (decreased fat mass and BMI and increased hydration), ultimately counteracting muscle decline. BMI: body mass index; OMWW-OL: olive mill wastewater enriched in polyphenols; PSH: plasma sulfhydryl groups; ROS: reactive oxygen species; SMM: skeletal muscle mass; TEAC: Trolox equivalent antioxidant capacity; %MM: percentage of muscle mass; ↓: decrease; ↑: increase. Solid arrows indicate direct involvement, whereas dotted arrows indicate secondary or downstream consequences.

The mechanism behind the observed stability of muscle mass and improved hydration status following OMWW-OL supplementation may be centered on muscle cell mitochondria function. Polyphenols contained in OMWW-OL are known to reduce oxidative stress by limiting ROS production. Since mitochondria are both a major source and target of ROS, oxidative stress leads to mitochondrial damage, including alterations in mitochondrial DNA, impaired oxidative phosphorylation, and reduced ATP production. This mitochondrial dysfunction can further trigger inflammatory pathways, establishing a vicious cycle between oxidative stress and inflammation. In skeletal muscle, which is highly dependent on mitochondrial energy metabolism, these alterations contribute to reduced muscle protein synthesis and increased degradation. By attenuating oxidative stress, OMWW-OL may preserve mitochondrial function, thereby supporting energy production and muscle integrity. In parallel, improved hydration status may further enhance mitochondrial efficiency, as adequate cellular water content is essential for optimal bioenergetic processes. Together, these mechanisms provide a coherent biological framework linking polyphenol intake to the attenuation of muscle decline (Figure 6).

This study has several limitations that should be carefully considered when interpreting the findings. The single-arm design and absence of a placebo control group preclude causal inference and substantially limit the ability to distinguish the effects of supplementation from natural temporal variability, dietary influences, or seasonal factors. As such, the results should be interpreted as exploratory and hypothesis-generating. The relatively small sample size and short follow-up duration reduce statistical power and limit the assessment of the sustainability of the observed effects. The use of a per-protocol analysis without imputation of missing data, together with participant attrition, may introduce bias and further constrain the generalizability of the results. In addition, the evaluation of multiple endpoints without formal adjustment for multiple comparisons raises the possibility of type I error inflation; therefore, nominal *p*-values should be interpreted with caution. Furthermore, body composition was assessed using bioelectrical impedance analysis, which, although validated under standardized conditions, is inherently sensitive to hydration status. The modest changes observed may therefore reflect measurement variability rather than true biological effects. Moreover, the concurrent improvement in hydration indices may confound the interpretation of lean mass estimates, potentially leading to circular inference. The lack of functional outcomes, such as muscle strength or physical performance measures, limits the ability to draw conclusions regarding clinically meaningful muscle health. The study provides limited mechanistic insight into how OMWW-derived polyphenols may influence muscle metabolism. Although changes in antioxidant markers (e.g., protein thiols and total antioxidant capacity) were observed, no direct assessment of oxidative stress modulation, inflammatory pathways, or mitochondrial function was performed. Additionally, the absence of detailed dietary assessment or control represents an important limitation, given the key role of nutritional factors, particularly protein intake, in muscle maintenance. Finally, the study population consisted of middle-aged to older adults with metabolic risk factors but with no evidence of muscle decline at baseline. Therefore, the findings may not be generalizable to populations with established sarcopenia or more severe metabolic impairment, limiting the clinical applicability of the results.

Nonetheless, the consistency of trends across multiple interrelated domains, including muscle mass indices, fat mass reduction, hydration status, and oxidative biomarkers, provides a coherent signal that warrants further investigation. Future studies should incorporate randomized placebo-controlled designs, larger sample sizes, longer follow-up, and standardized measurement conditions, as well as functional endpoints to determine clinical relevance. Further exploration in populations with established muscle impairment may also be warranted, although extrapolation to conditions such as cancer-associated sarcopenia should be approached cautiously. Moreover, a broader set of endpoints should be assessed to more comprehensively characterize muscle health beyond body composition alone. In particular, the inclusion of functional parameters such as handgrip strength, gait speed, and chair stand tests would allow assessment of clinically meaningful outcomes. Measures of muscle quality, including imaging-derived muscle density or ultrasound-based echo intensity, should also be considered. Given the proposed mechanism of action, evaluation of mitochondrial function (e.g., ATP production, mitochondrial respiration, and biogenesis markers such as PGC-1α) would provide important mechanistic insights. In addition, while previous work has primarily focused on circulating oxidized LDL and selected metabolomic ratios, future studies should expand the assessment of oxidative stress and inflammation to include more sensitive and pathway-specific biomarkers. These may encompass markers of lipid peroxidation (e.g., F2-isoprostanes), protein oxidation (e.g., protein carbonyls), and DNA oxidative damage (e.g., 8-hydroxy-2′-deoxyguanosine), alongside endogenous antioxidant system components such as glutathione redox status (GSH/GSSG) and catalase activity. Similarly, a more comprehensive inflammatory profiling could include high-sensitivity CRP, TNF-α, IL-6, and IL-1β, as well as emerging markers of chronic low-grade inflammation such as MCP-1 and adhesion molecules (e.g., ICAM-1 and VCAM-1), which may better capture subtle systemic responses not addressed in prior analyses. Additionally, more detailed hydration assessments, including intracellular and extracellular water distribution, could further elucidate the relationship between hydration status and muscle physiology, extending previous observations of improved total hydration. Finally, the integration of markers of muscle protein turnover (e.g., circulating amino acid kinetics, 3-methylhistidine, or ubiquitin–proteasome pathway markers) and patient-centered outcomes would contribute to a more holistic evaluation of muscle maintenance and vulnerability.

## 5. Conclusions

In this secondary, hypothesis-driven re-analysis, short-term supplementation with a standardized olive mill wastewater (OMWW) polyphenol extract (Oliphenolia^®^, OMWW-OL) was associated with consistent directional trends toward maintenance of skeletal muscle mass indices, reduction in fat mass, improved hydration status, and enhanced antioxidant reserve in adults at risk of metabolic syndrome. Although the observed effects were modest and not uniformly statistically significant, the overall directional consistency may suggest a biologically plausible supportive role that requires confirmation in controlled studies. Notably, improvements in muscle-related parameters were accompanied by reductions in body weight and BMI, outcomes generally considered as highly desirable in nutraceutical supplementation for individuals with pre-metabolic syndrome. These changes were observed alongside improved hydration metrics, although these findings should be interpreted cautiously given the influence of hydration on BIA-derived measures, suggesting a possible favorable metabolic adaptation rather than lean tissue loss. These effects are consistent with the known antioxidant and anti-inflammatory properties of OMWW-derived polyphenols, which may contribute to modulation of key pathways involved in muscle decline.

Overall, these findings support further investigation of OMWW-derived polyphenols, rather than establishing efficacy, as a nutraceutical strategy for the early prevention of muscle mass decline, particularly in populations at risk of metabolic syndrome. Any extension of these findings to other clinical contexts, including cancer- or cardiovascular-associated sarcopenia, remains speculative and should be addressed in specifically designed studies. Larger and adequately powered trials are needed to confirm efficacy, elucidate underlying mechanisms, and determine long-term clinical relevance in metabolic syndrome and other conditions characterized by muscle vulnerability.

## Figures and Tables

**Table 1:** Polyphenol composition of a typical batch of Oliphenolia^®^ (per 2 × 25 mL daily serving).

Polyphenols	Amount per 50 mL
Hydroxytyrosol	82.50 mg
Tyrosol	10.50 mg
Verbascoside	32.50 mg
p-Coumaroyl secoiridoids	6.50 mg
Other polyphenols	318.50 mg
Total polyphenols	450.55 mg

## Data Availability

The original contributions presented in this study are included in the article. Further inquiries can be directed to the corresponding author.
